# La mélorhéostose

**DOI:** 10.11604/pamj.2014.19.314.5328

**Published:** 2014-11-21

**Authors:** Kawtar Nassar, Ouafa Mkinsi

**Affiliations:** 1 Service de Rhumatologie, Centre Hospitalier Universitaire, Casablanca, Maroc

**Keywords:** mélorhéostose, radiologie, ostéocondensation, melorheostosis, radiology, osteocondensation

## Image en medicine

La mélorhéostose est une dysplasie osseuse sclérotique progressive. Son incidence est estimée à 0,9 par million d'habitants. C'est une maladie congénitale, décrite par A. Léri et L. Joanny en 1922. Elle reste méconnue du fait de sa rareté. Elle débute insidieusement dans l'enfance, et touche les os longs des membres inférieurs. Progressivement s'installe une raideur articulaire et une impotence fonctionnelle. La douleur, la raideur et les déformations articulaires constituent le profil évolutif de la maladie. Certaines hypothèses évoquent un émutation du gène du mésoderme et du gène LEMD3. Son diagnostic est posé sur les données caractéristiques des radiographies standards. L'histologie est inutile en cas de forme typique. Nous en rapportons un cas chez une patiente âgée de 44 ans, présentant depuis l’âge de 12 ans, des gonalgies droites mixtes et intermittentes, soulagées par traitements symptomatiques. L’évolution était marquée par la reprise de la même symptomatologie occasionnant progressivement une boiterie à la marche, et apparition d'une tuméfaction et incurvation de la cuisse du même côté. L'examen physique retrouvait; une attitude du membre inférieur droit en rotation externe et abduction, une tuméfaction de la cuisse et une raideur de la hanche et du genou droit. Le bilan biologique était normal. Les radiographies standard retrouvaient de multiples opacitéslongilignes en «coulées de bougie» s’étendant le long de la corticale fémorale et tibio-peronièredroite et ossifications des tissus mous para articulaire et osseux ( A et B), confirmées au scanner (C) et à la scintigraphie osseuse, fixant les zones d'ostéoformation (D). La patiente était mise sous antalgiques et bisphosphonates orale. Le diagnostic différentiel se pose avec l'ostéosarcome paraostéal, la myosite ossifiante, l'hématome calcifié ou une ossification des parties molles. Le traitement fait appel aux antalgiques, bisphosphonates, colchicine, vasodilatateurs, corticothérapie. La chirurgie en association avec une rééducation sont proposées pour limiter la maladie.

**Figure 1:**
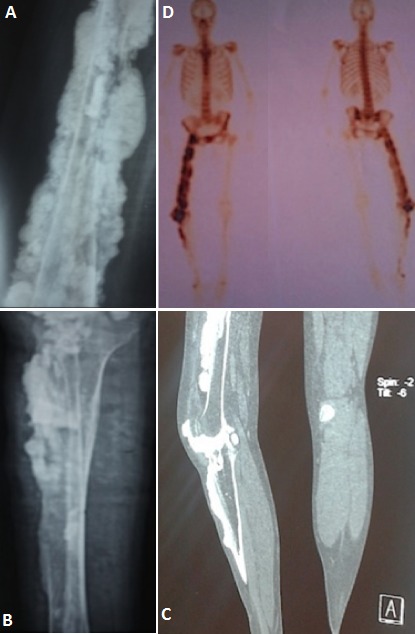
(A) radiographie du fémur droit, retrouve des opacités longilignes en «coulées de bougie» s’étendant le long de la corticale fémorale droite avec de multiples foyers d'ostéoscléroses nodulaires et ossification des tissus mou para articulaires et osseux; (B) radiographie standard de la jambe droite, en faveur d'opacitéslongilignes tibio-peronière associées à des foyers d'ostéoscléroses nodulaires; (C) scanner des membres inférieurs, montre des hypercondensations linéaires en «coulées de bougie», étendues de la corticale du fémur droit, bien limitées, parallèle au grand axe de l'os, avec comblement par endroit de la médullaire; (D) cintigraphie osseuse, hyperfixation osseuse le long du fémur droit et de l'extrémité supérieure de la jambe du même côté

